# Healthy assemblages of *Isidella elongata* unintentionally protected from trawling offshore of Asinara Island (northwestern Sardinia, NW Mediterranean Sea)

**DOI:** 10.1038/s41598-024-63652-1

**Published:** 2024-06-04

**Authors:** M. Angiolillo, B. Di Lorenzo, A. Izzi, M. Giusti, O. Nonnis, A. Pazzini, B. Trabucco, L. Tunesi

**Affiliations:** Istituto Superiore per la Protezione e Ricerca Ambientale (ISPRA), Via Vitaliano Brancati, 60, 00144 Rome, Italy

**Keywords:** Ecology, Environmental sciences, Ocean sciences, Marine biology

## Abstract

Deep-sea coral assemblages are marine biodiversity hot spots. Because of their life history traits, deep-sea corals are highly vulnerable to the impacts of human activities such as fishing. The critically endangered “bamboo coral” *Isidella elongata* is a key structuring species of deep muddy bottoms that is susceptible to habitat destruction, particularly from trawling. A shallow population of this species was recently discovered by a multibeam and ROV survey offshore of the Asinara Island marine protected area (MPA) (northwestern Sardinia, NW Mediterranean Sea). This vulnerable marine assemblage has been found under healthy conditions at depths ranging from 110 to 298 m. *Isidella elongata* occurs on a muddy seafloor locally characterised by boulders associated with black coral species (*Parantipathes larix* and *Antipathes dichotoma*). The lush colonies of *I. elongata* seem to be related to natural protection from bottom trawling activity; nevertheless, the presence of lost fishing artisanal nets has been observed in the study area. These structuring species are indicators of vulnerable marine ecosystems, and their conservation is essential for preserving marine biodiversity. Therefore, enlarging the perimeter of the Asinara Island MPA into its deeper western waters is suggested to ensure the protection of these valuable and vulnerable marine ecosystems.

## Introduction

The bamboo coral *Isidella elongata* (Esper, 1788) is a two-dimensional candelabrum-shaped octocoral, and it is considered a key component of deep marine ecosystems. It is distributed in the Mediterranean Sea and in the adjacent Gulf of Cadiz and northern Morocco^1–3^. The occurrence of this near-endemic species within the Mediterranean basin has been recorded both in the western^4–8^ and central regions^9–11^, whereas few data come from the Adriatic Sea and other seas of the eastern basin^4,12^. The bathymetric range of this species extends from 115 m down to 1656 m depth^4,11,13,14^, and it is found mainly on a slope gradient of less than 5%^15–17^. It can form important *facies* (sensu Pérès and Picard^15^) in the middle horizon of bathyal soft bottoms, mainly at depths greater than 500 m^4,18,19^. The height of its colonies hardly exceeds 80 cm in the Mediterranean Sea^14,20^.

The bamboo coral plays multiple important ecological roles in the deep sea. It is a habitat-forming species able to form extensive aggregations of colonies that can increase the heterogeneity and three-dimensional complexity of the habitat (Ref.^3,21^; and references therein). As a sessile species, it performs a structural role by providing a secondary biological hard substratum for some epibiont species, such as *Amphianthus dohrnii *(Koch, 1878) and *Anamathia rissoana* (Roux, 1828). Additionally, this species serves as a spawning substratum and nursery ground for cephalopods and sharks (i.e., the veined squid *Loligo forbesii* Steenstrup, 1856 and the blackmouth catshark *Galeus melastomus* Rafinesque, 1810)^21^. An increase in local biomass within bamboo coral *facies* and their canopies can influence the availability of resources, thus promoting bentho-pelagic food webs. This, in turn, attracts benthic invertebrates and leads to high concentrations of commercially valuable species seeking refuge, feeding grounds, and spawning areas^10,14,21–23^. Several important commercial species, such as the red shrimp *Aristeus antennatus* (Risso, 1816), the giant red shrimp *Aristaeomorpha foliacea* (Risso, 1827), the deep-water rose shrimp *Parapenaeus longirostris* (Lucas, 1846), the Norway lobster *Nephrops norvegicus* (Linnaeus, 1758) and many fish species, such as *Merluccius merluccius* (Linnaeus, 1758), *Micromesistius poutassou* (Risso, 1827), *Phycis blennoides* (Brünnich, 1768), *Lepidorhombus boscii* (Risso, 1810), *Helicolenus dactylopterus* (Delaroche, 1809), and *G. melastomus*, are known to co-occur within the *I. elongata facies* (Ref.^3,9,10,24–26^, and references herein). It has been noted that some species, not exclusively associated with the *Isidella facies*, exhibit a notably greater biomass and/or size within these *facies* than in the areas where they are absent^3,10,21^. This observation highlights the potential role of bamboo coral habitat as a breeding and/or feeding area. Nevertheless, the *I. elongata facies* is affected by several stressors and is currently considered a sensitive habitat heavily impacted by deep-sea fisheries^4,5,7,11,14^. The co-occurrence of *I. elongata* populations with species of high commercial value makes them an indirect target of bottom trawling activities. This fishing practise can have devastating impacts on pristine gorgonian forests by removing and breaking colonies (e.g.,^7,18,21,23^, and references therein). Bamboo coral was once considered very abundant in the deep Mediterranean Sea^13,27^, but currently, it occurs scattered over localised areas at very low abundances^28^. This species has likely already disappeared from many trawled bottoms in the western Mediterranean Sea, including the Catalan Sea, the Balearic Sea, the canyons of the Gulf of Lions, the Ligurian Sea, and the Strait of Sicily (e.g.,^5,11,18,21,23^), where a notable decline in coral bycatch was observed, mainly during the 1970s-1980s. Even bottom fishing long-lines are recognised as a significant threat to bamboo coral colonies because they often lead to entanglement events, damaging the living tissues of corals, eradicating the colonies, or breaking the coral branches^3,10,13,29,30^. Moreover, indirect effects such as sediment runoff and trawling-induced sediment displacement can increase turbidity near the bottom in *Isidella* habitats, facilitating the clogging of polyps and thereby constraining their filter feeder functionality^3,31,32^.

A slow growth rate, a long-life span (~ 400 years), and a limited dispersal capability determine the low resilience of this species and, consequently, its very low potential for recovery^3,13,33^.

Several agreements recognised the importance of conserving *I. elongata* and the *facies* it creates. This species is considered ‘‘Critically Endangered’’ for the International Union for Conservation of Nature (IUCN) Red List of Threatened Species, both in the Mediterranean Sea and in Italian waters^34,35^; it is considered an indicator species of Vulnerable Marine Ecosystems (VMEs) by the General Fisheries Commission for the Mediterranean Sea (GFCM)^36^; it is a key facies of ‘‘Deep Water Engineering benthic invertebrate assemblages” listed under the ‘‘Dark Habitats Action Plan’’ of the Barcelona Convention^37^; and it is listed in Annex II of the Barcelona Convention Protocol concerning Specially Protected Areas and Biological Diversity in the Mediterranean because it is recognised as an “endangered or threatened species”^38^. Finally, the Marine Strategy Framework Directive (MSFD, 2008/56/EC) recognises that several fragile deep-sea habitats need protection because the removal of their species can also affect some other species that live in association with corals.

However, despite the current limited distribution of these fragile bathyal muddy-bottom *facies* in the Mediterranean Sea, some populations of bamboo coral have been indirectly preserved. This occurs in some areas where trawling is avoided due to the presence of submarine pipelines or due to the nature of the seabed itself (such as deep hard bottoms, coral banks, and canyon flanks)^5,9,21^.

The assessment of species distribution, population structure, conservation status, and understanding of the ecological interactions between VMEs and environmental and human constraints is of paramount importance for obtaining valuable information to establish adequate management and conservation plans, especially for vulnerable and endangered species.

Off the coast of the Asinara Island marine protected area (MPA) in western Sardinia (Mediterranean Sea), a population of *I. elongata* was investigated through a multibeam echosounder and remotely operated vehicle (ROV) survey describing it as a healthy population.

The main aims of this study are (1) to map the occurrence of *I. elongata* on the explored western offshore Asinara Island MPA, (2) to quantify the density and population-size distribution of this species, (3) to describe the associated megabenthic fauna and, finally, (4) to discuss the observed distribution pattern in relation to the environmental setting of the study area. Furthermore, the main anthropogenic threats affecting the study area are also reported. The results are discussed considering existing knowledge on this species in the Mediterranean Sea.

## Results

### Habitat characterisation

**Figure 1:**
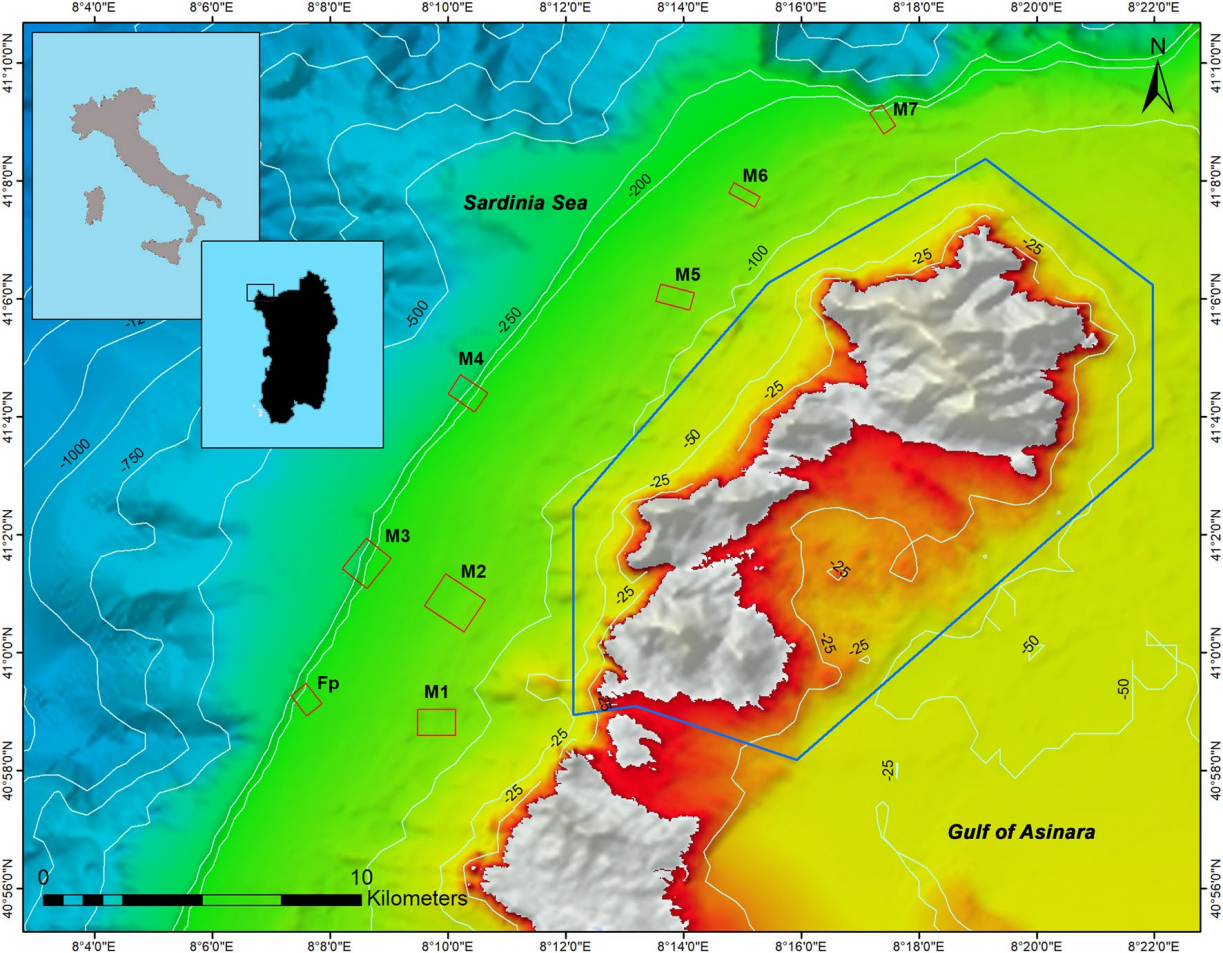
Investigated area. Upper left insets: Italy and the Sardinia region. The red rectangles indicate the sites where high-resolution multibeam surveys were carried out (except for Fp) and the locations of the eight remotely operated vehicle (ROV) dives (M1–M7, Fp). The blue polygon indicates the perimeter of the Asinara Island MPA. Background bathymetry data were obtained from EMODnet (https://emodnet.ec.europa.eu/en/bathymetry). The map was generated using ESRI's ArcGIS 10.3 for Desktop software (http://www.esri.com), version 10.3.0.4322.

The investigated study area is located within a complex topographic region. It is primarily characterised by a sandy bottom with scattered boulders within the mesophotic range (66–150 m), by a soft bottom up to 250 m and by a rocky escarpment that extends from 250 to 307 m depth. Eight sites offshore of the Asinara Island MPA were investigated by means of a multibeam echosounder and a ROV (Fig. 1). The eight ROV dives produced a total of 6 h and 53 min of footages. The transect lengths ranged between ∼ 400 and 900 m, covering a total survey distance of ~ 5 km, and a depth range of 66–307 m (Table 1). Overall, hard substratum covered up to 52.4% of the explored sea bottom (Table 1).

**Table 1:** Summary of the technical data for the remotely operated vehicle (ROV) dives performed in the study area, including the data, dive code, geographical coordinates, depth range (m), total time, total length (m), number of sampling units (SUs), and percentage (%) of SUs with hard substrata.

Data	Dive code	Latitude start	Longitude start	Depth range (m)	Total time	Transect length (m)	SU (no.)	% of SU with hard substrata
25/07/2022	Fp	40.98696	8.125695	107–196	00:37:34	348	52	9.6
01/08/2022	M1	40.98132	8.162838	74–130	00:45:22	640	58	36.2
01/08/2022	M2	41.01393	8.167115	66–147	01:07:10	750	162	56.2
01/08/2022	M3	41.02215	8.143262	130–240	01:06:55	760	160	48.8
02/08/2022	M4	41.07402	8.169812	150–307	00:47:35	800	136	7.4
02/08/2022	M5	41.10105	8.228472	79–134	00:54:50	850	159	73.6
02/08/2022	M6	41.13103	8.248923	103–150	00:44:44	875	113	86.7
02/08/2022	M7	41.14878	8.290821	118–180	00:49:30	436	105	71.4

The investigated western side of Asinara Island was not subjected to intense trawling, as evidenced by the Vessel Monitoring System (VMS) and Automatic Identification System (AIS) data tracking of trawling vessels (i.e.,^39^; Fig. S1); however, artisanal fishing activities (i.e. trammel nets, longlines, and traps) were observed.

### General megafaunal characteristics

**Figure 2:**
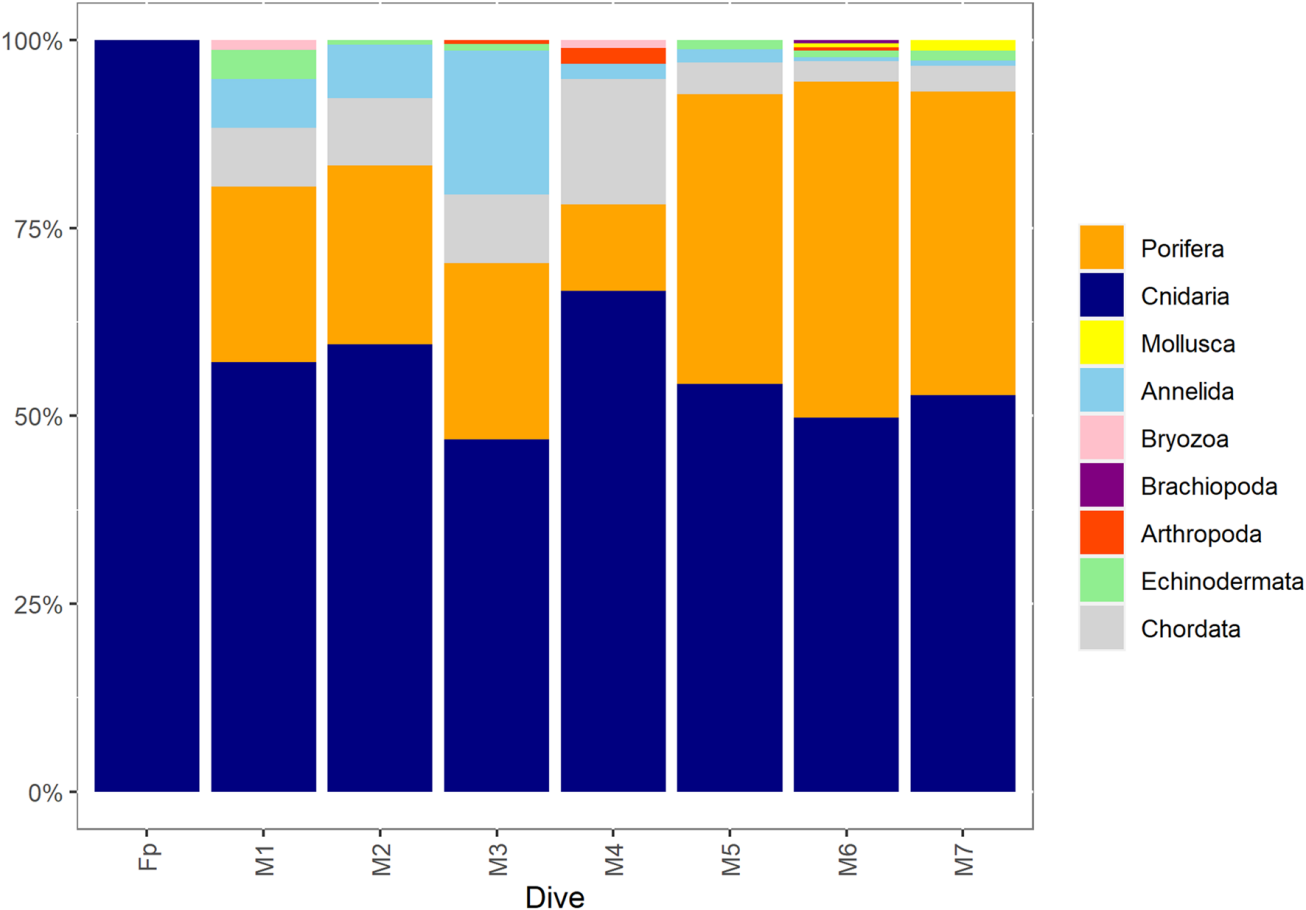
Megabenthic species richness (%) per phylum in each dive.

**Figure 3:**
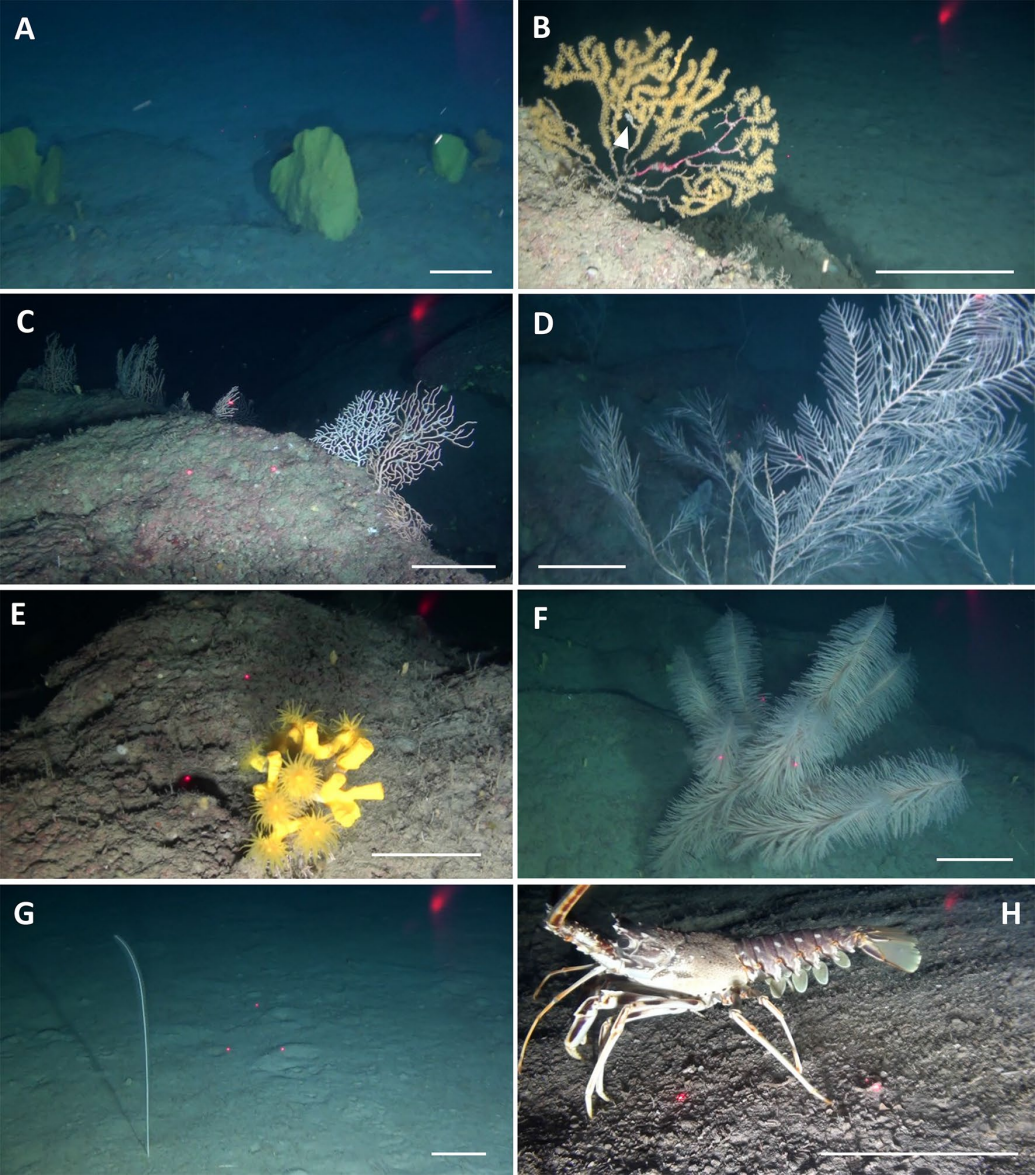
Megafauna in the study area. (**A**) *Phakellia* sp.; (**B**) *Paramuricea hirsuta* with Tritoniidae nd. specimen (white arrow); (**C**) *Eunicella cavolini* and *E. verrucosa*; (**D**) *Callogorgia verticillata*; (**E**) *Dendrophyllia cornigera*; (**F**) *Parantipathes larix*; (**G**) *Funiculina quadrangularis*; (**H**) *Palinurus elephas*. Scale bar: 15 cm.

**Figure 4:**
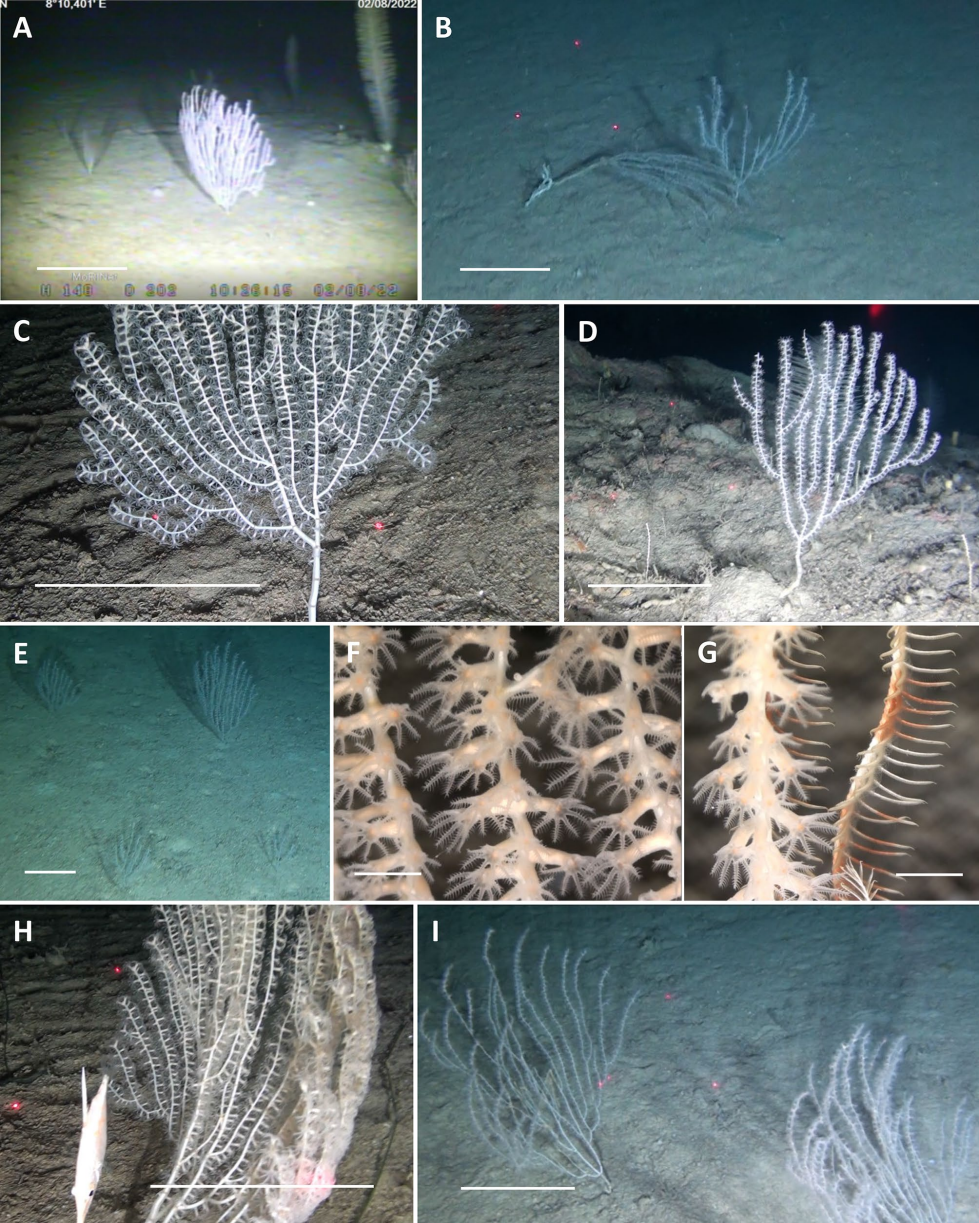
*Isidella elongata facies*. (**A**) *I. elongata* and *Parantipathes larix*; (**B**–**E**) Colonies of *I. elongata*; (**F**) Close-up image of *I. elongata* polyps; (**G**) Close-up image of *Leptometra phalangium* branches on an *I. elongata* colony; (**H**) *Macroramphosus scolopax* swimming near a bamboo coral colony; (**I**) Healthy and tall colonies of *I. elongata*. Scale bar (**A**–**E**, **H**–**I**): 15 cm; Scale bar (**F**–**G**): 0.5 cm.

The mesophotic and bathyal bottom of the western side of Asinara Island hosted a plurispecific megabenthic assemblage, with a total of 65 different taxa observed (Table 2). Cnidaria (43.1%), Chordata (21.5%) and Porifera (13.8%) were the most common Phyla, followed by Echinodermata (9.2%) (Table 2, Figs. 2, 3). However, some species of sponges characterized by encrusting habitus and small size were observed but not identified, so this phylum was partially underestimated. The scattered boulders in the mesophotic range were covered by encrusting sponges and dwelled by massive sponge [e.g. *Pachastrella monilifera* Schmidt, 1868*, Poecillastra compressa* (Bowerbank, 1866)*, Phakellia* sp. (Fig. 3A)], bryozoans, serpulids, molluscs, and gorgonians [e.g., *Swiftia dubia* (Thomson, 1929), *Bebryce mollis* Philippi, 1842, *Paramuricea hirsuta* Gray, 1857 (Fig. 3B), *Eunicella cavolini* (Koch, 1887) and *E. verrucosa* (Pallas, 1766) (Fig. 3C), *Callogorgia verticillata* (Pallas, 1766) (Fig. 3D), and *Viminella flagellum* (Johnson, 1863)]. Healthy colonies of scleractinian *Dendrophyllia cornigera* (Lamarck, 1816) (Fig. 3E), the antipatharian *Antipathes dichotoma* (Pallas, 1766), *Leiopathes glaberrima* (Esper, 1788), and, mainly, *Parantipathes larix* (Esper, 1788) (Fig. 3F) were also observed (Table 2). The plateau and the muddy bottom around the boulders were characterised by an important *facies* of the bamboo coral *Isidella elongata* (Fig. 4) and several colonies of *Funiculina quadrangularis* (Pallas, 1766) (Fig. 3G). The escarpment was characterised by several sponges, gorgonians (*P. hirsuta* and *C. verticillata*), and black and soft corals. Nine of the species found here are classified as vulnerable to critically endangered by the IUCN, including the common spiny lobster *Palinurus elephas* (Fabricius, 1787) (Table 2, Fig. 3H).

**Table 2:** Species list showing classification by conservation status according to the Red List of Threatened Species (IUCN).

Phylum/class	Taxon	IUCN	Phylum/class	Taxon	IUCN
Porifera			Mollusca		
	Porifera ind		Cephalopoda	Octopodidae	
Demospongiae	*Phakellia* spp.		Gastropoda	Tritoniidae	
	*Haliclon*a spp.		Annelida		
	*Axinella* spp.		Polychaeta	*Bonellia viridis*	
	*Axinella damicornis*			*Filograna implexa*	NE
	**Pachastrella monilifera**	**VU (Italy)**	Bryozoa		
	**Poecillastra compressa**	**VU (Italy)**	Gymnolaemata	*Reteporella* spp.	
	*Rhyzaxinella* sp.		Brachiopoda		
Hexactinellida	*Farrea* sp.		Rhynchonellata	*Gryphus vitreus*	
Cnidaria			Arthropoda		
Octocorallia	Alcyonacea ind		Malacostraca	*Anamathia rissoana*	
	*Alcyonium acalue*	LC (Med)		**Palinurus elephas**	**VU**
	*Alcyonium coralloides*	LC (Med)	Echinodermata		
	*Chironephthya mediterranea*		Asteroidea	Asteroidea	
	*Bebryce mollis*	DD (Med)		*Peltaster placenta*	
	*Callogorgia verticillata*	NT (Med)	Crinoidea	*Leptometra phalangium*	
	***Corallium rubrum***	**EN (Med)**	Echinoidea	Cidaridae	
	*Eunicella cavolini*	NT (Med)		*Echinus melo*	
	*Eunicella verrucosa*	NT (Med)	Holoturidea	*Holoturia tubulosa*	
	Malacalcyonacea ind		Chordata		
	*Paramuricea hirsuta*	LC (Med)	Ascidiacea	*Halocynthia papillosa*	
	*Paramuricea macrospina*	DD (Med)	Teleostei	*Anthias anthias*	LC (Med)
	*Spinimuricea klavereni*	DD (Med)		*Acantholabrus palloni*	LC (Med)
	*Swiftia dubia*	DD (Italy)		*Aulopus filamentosus*	LC (Med)
	*Villogorgia bebrycoides*	DD (Med)		*Chlorophthalmus agassizi*	LC (Med)
	*Viminella flagellum*	NT (Med)		*Helicolenus dactylopterus*	LC (Med)
	***Funiculina quadrangularis***	**VU (Med)**		*Lappanella fasciata*	LC
	***Isidella eleongata***	**CR (Med)**		*Lepidorhombus* spp.	LC
	*Kophobelemnon stelliferum*	LC (Med)		*Macroramphosus scolopax*	LC (Med)
	***Pennatula rubra***	**VU (Med)**		*Serranus cabrilla*	LC (Med)
	*Virgularia mirabilis*	LC (Med)		Macrouridae	
Hexacorallia	*Amphianthus dohrnii*	NE		*Scorpaena* sp.	LC
	*Cerianthus* spp.			*Zeus faber*	LC (Med)
	***Dendrophyllia cornigera***	**EN (Med)**		*Trachinus draco*	LC (Med)
	*Antipathella subpinnata*	NT (Med)			
	*Antipathes dichotoma*	NT (Med)			
	***Leiopathes glaberrima***	**EN (Med)**			
	*Parantipathes larix*	NT (Med)			

*CR* critically endangered, *EN* endangered, *VU* vulnerable, *NT* near threatened, *LC* least concern, *DD* data deficient, *NE* not evaluated.

Significant values (CR, EN, and VU) are in [bold].

**Figure 5:**
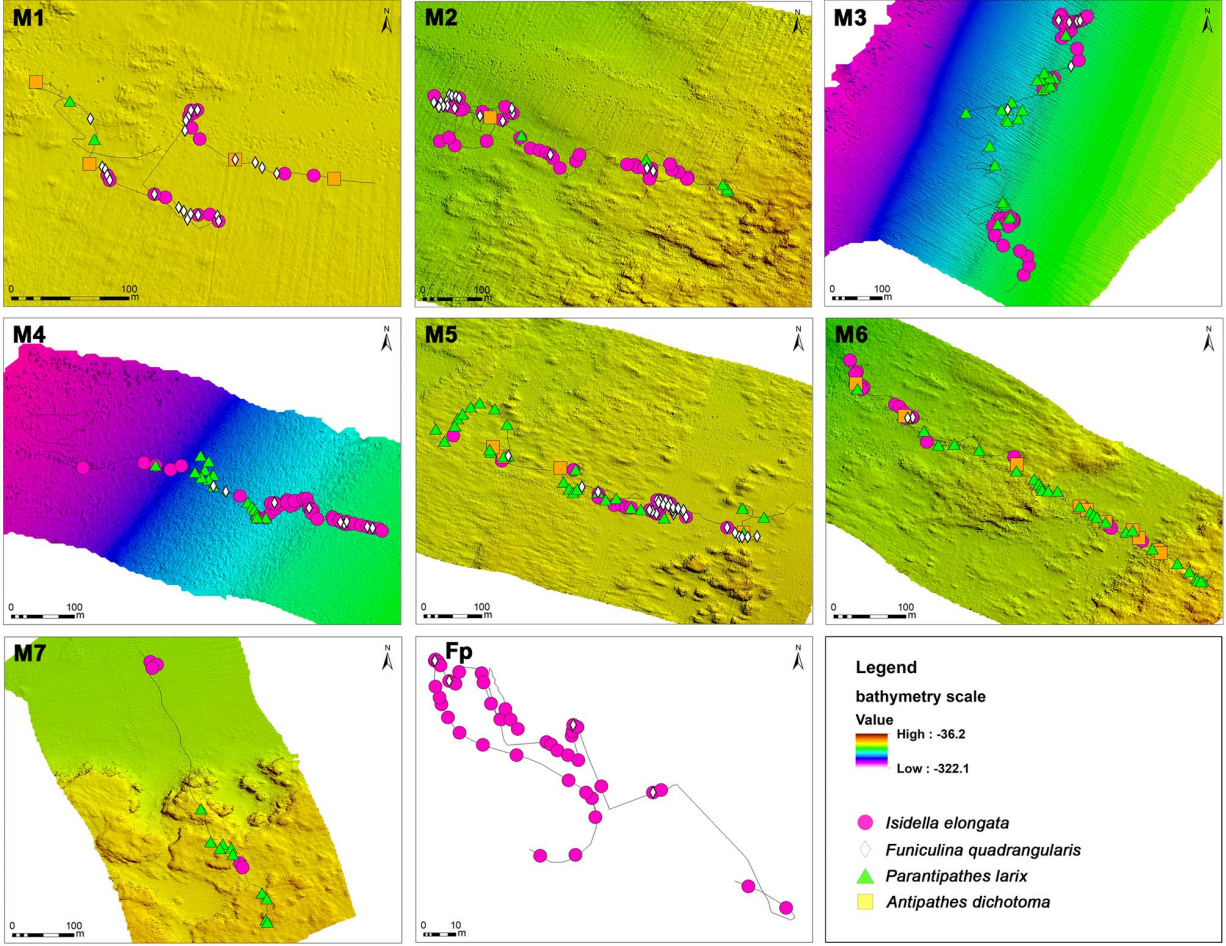
High-definition bathymetric maps (except for dive Fp) with eight explorative remotely operated vehicle (ROV) transects (M1–M7, Fp) and the spatial location of the most relevant megabenthic species identified in the area.

Among the habitat-forming species observed, the bamboo coral *I. elongata* (Fig. 4), the gorgonian *P. hirsuta*, the black coral *P. larix,* and the sea pen *F. quadrangularis* were the most abundant species (Table 3, Fig. 5), representing 70.2%, 7.67%, 7.43%, and 6.6%, respectively, of all the observed organisms.

**Table 3:** Number of colonifes (no.), occupancy (% of sampling units occupied by a species), and density (no. of colonies m^−2^ ± *se*) of the most relevant megabenthic species.

	M1	M2	M3	M4	M5	M6	M7	Fp
*Isidella elongata*								
No.	23	71	239	354	53	48	7	660
%	27.6	22.2	30.6	31.6	14.5	21.2	4.8	82.7
Col. m^−2^ ± *se*	0.08 ± 0.02	0.09 ± 0.02	0.30 ± 0.05	0.52 ± 0.09	0.07 ± 0.02	0.08 ± 0.02	0.01 ± 0.01	2.54 ± 0.26
Depth range (m)	120–125	110–141	145–203	190–298	125–130	132–149	125–155	110–192
*Funiculina quadrangularis*								
No.	42	22	6	11	49	2	0	4
%	37.9	11.1	3.8	7.4	17.0	1.8	–	7.7
Col. m^−2^ ± *se*	0.14 ± 0.03	0.03 ± 0.01	0.01 ± 0.00	0.01 ± 0.00	0.06 ± 0.01	< 0.01	–	0.02 ± 0.01
Depth range (m)	120–125	130–140	193–221	191–250	123–128	140–141	–	172–187
*Paramuricea hirsuta*								
No.	8	43	12	2	40	27	12	6
%	10.3	16. 7	3.7	1.5	12.5	20.3	6.7	5.7
Col. m^−2^ ± *se*	0.03 ± 0.01	0.05 ± 0.01	0.01 ± 0.01	< 0.01	0.05 ± 0.01	0.05 ± 0.01	0.02 ± 0.01	0.02 ± 0.01
Depth range (m)	81–127	119–139	195–216	238–261	94–129	126–145	121–125	186–191
*Callogorgia verticillata*								
No.	4	35	6	2	1	1	0	3
%	5.2	9.9	3.1	0.7	0.6	0.9	–	3.8
Col. m^−2^ ± *se*	0.01 ± 0.01	0.04 ± 0.01	0.01 ± 0.01	< 0.01	< 0.01	< 0.01	–	0.01 ± 0.01
Depth range (m)	81–127	128–139	202–214	211–211	123–123	138–138	–	186–191
*Parantipathes larix*								
No.	3	6	28	28	35	37	17	0
%	3.4	3.1	15.0	11.0	17.6	23.0	9.5	–
Col. m^−2^ ± *se*	0.01 ± 0.01	0.01 ± 0.00	0.04 ± 0.01	0.04 ± 0.01	0.04 ± 0.01	0.07 ± 0.01	0.03 ± 0.01	–
Depth range (m)	121–123	128–135	188–238	234–286	120–131	125–143	121–126	–
*Antipathes dichotoma*								
No.	4	1	0	0	4	10	0	0
%	6.9	0.6	–	–	2.5	8.8	–	–
Col. m^−2^ ± *se*	0.01 ± 0.01	< 0.01	–	–	0.01 ± 0.00	0.02 ± 0.01	–	–
Depth range (m)	122–123	138–138	–	–	123–129	130–145	–	–
*Dendrophyllia cornigera*								
No.	4	8	19	0	6	14	13	0
%	6.9	3.7	8.8	–	3.8	8.8	9.5	–
Col. m^−2^ ± *se*	0.01 ± 0.01	0.01 ± 0.01	0.02 ± 0.01	–	0.01 ± 0.003	0.02 ± 0.01	0.02 ± 0.01	–
Depth range (m)	119–126	110–136	188–211	–	123–126	125–145	122–149	–

The black coral *P. larix* was widespread across all the dives (except for Fp dive), with tall and branched colonies occurring within the depth range of 120–286 m, and density values between 0.01 ± 0.04 and 0.07 ± 0.15 col. m^⁠−2^, up to 1 col. m^⁠⁠−2^.

The tall sea pen *F. quadrangularis* was recorded on a soft substratum with densities ranging between 0.01 ± 0.04 and 0.14 ± 0.22 col. m^⁠−2^, in the depth range of 120–250 m.

### *Isidella elongata* facies

**Figure 6:**
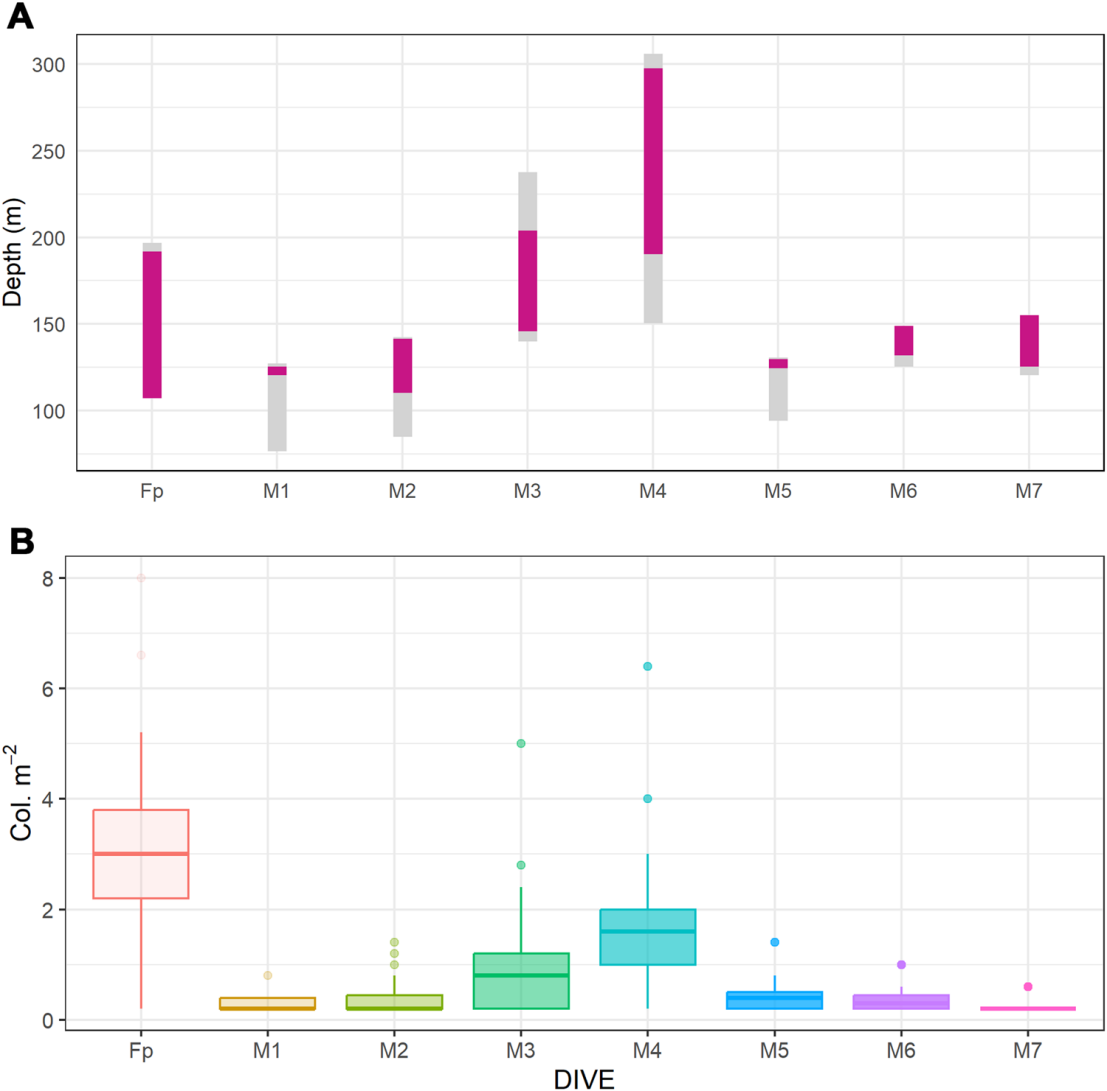
Bathymetric distribution and colony densities of *Isidella elongata*. (**A**) The grey rectangles represent the bathymetric range explored for each dive, and the fuchsia rectangles represents the bathymetric range where *I. elongata* was found. (**B**) Boxplot of the colony density of *I. elongata* in each dive: boxes indicate the first and third quartiles; bold lines indicate the median value; lines indicate the range between the minimum and maximum values; and dots indicate outliers.

All the exploratory dives performed revealed the presence of *I. elongata* in the study area (Table 3, Fig. 1). A total of 1453 colonies of this species was counted along all the video transects over an area of 5.45 km^2^ at depths ranging between 110 and 298 m (Figs. 5, 6A). The colonies were settled on a detritic sand and semi-consolidated muddy seafloor, sometimes proximal to hard substrata.

Bamboo coral occurred in 25% of the total sampling units, with high variation among the dives (Table 3, Fig. 6B). A greater occupancy and density of this species was recorded at the offshore sites (Fp, M3, M4), and 59% of the colonies were found at depths ranging from 180 to 200 m (Table 3). The average density ranged from 0.01 col. m^−2^ ± 0.07 and 2.54 col. m^−2^ ± 1.84, with a maximum value of 8 col. m^−2^ in the dive Fp (Table 3, Fig. 6B).

**Figure 7:**
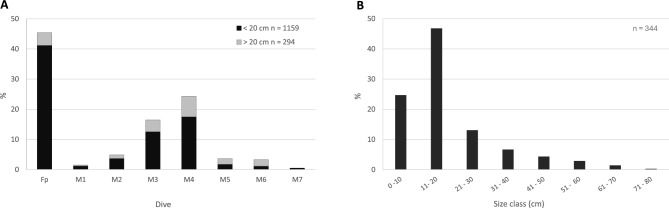
Population structure of *Isidella elongata* colonies. (**A**) Percentage of total small (< 20 cm height) and large (> 20 cm height) colonies at each site. (**B**) Size distribution of colony height on a subsample of *I. elongata* colonies in the study area.

Among all the colonies observed, only a few were large and tall (> 20 cm) and characterised by the typical candelabrum-shaped morphology, with a high number of branches and open polyps (Figs. 4, 7A). In contrast, most of the colonies (70%) were young and characterised by low numbers of branches and small sizes (< 20 cm). A subsample of 344 colonies was measured, and the colony size ranged from 5.6 to 71.3 cm. Analysis of the colony height for the whole study area, revealed asymmetrical, non-normal distributions of the size descriptor (Fig. 7B). Height distributions exhibited significant positive skewness and kurtosis (leptokurtic), displaying the highest peaks in the smaller classes (0–10 and 11–20 cm), and a long-tailed distribution featuring some large colonies.

**Figure 8:**
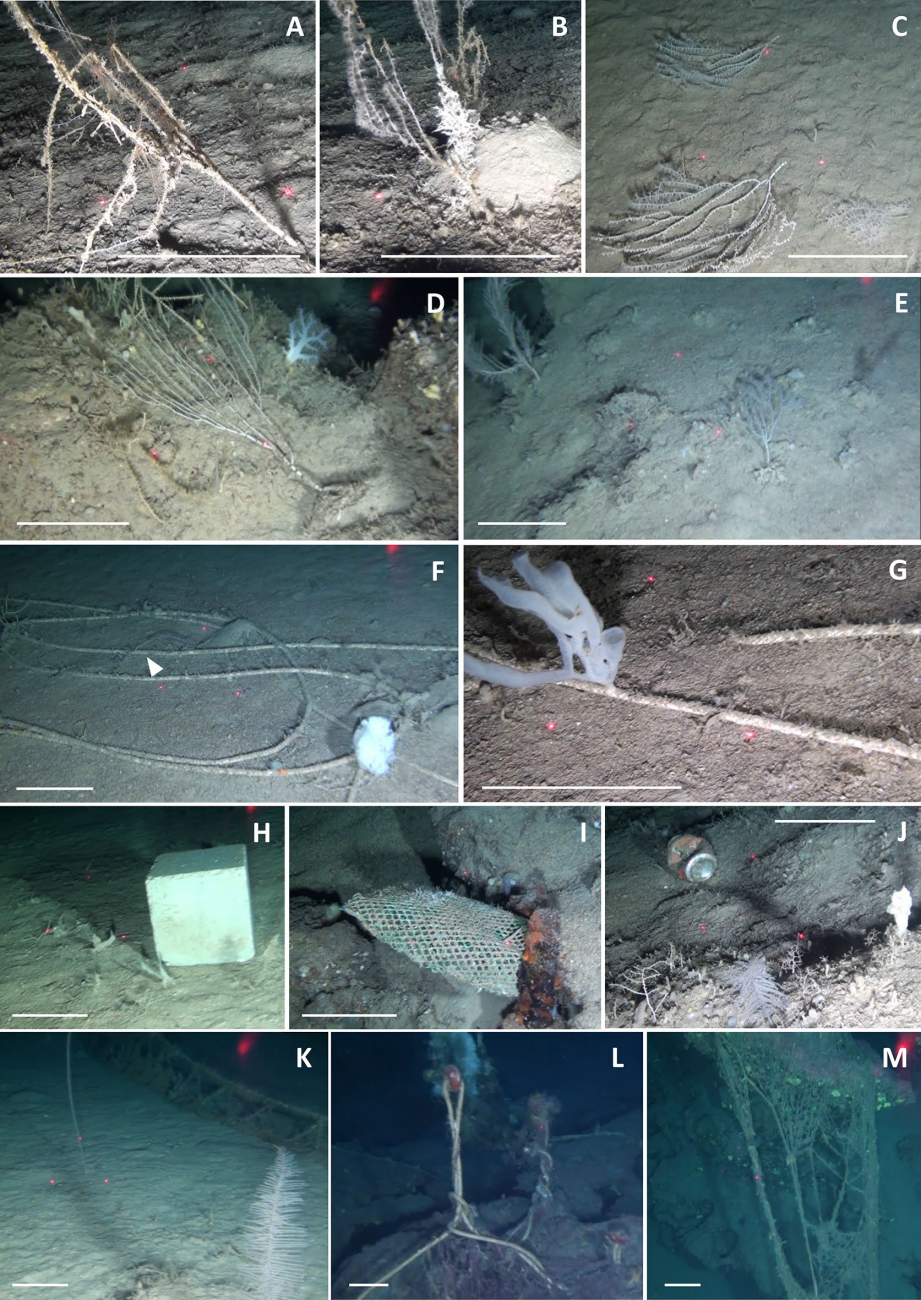
Anthropogenic impact in the study areas. (**A**,**B**) Epibionted *Isidella elongata* colonies; (**C**–**E**) Broken and eradicated colonies of *I. elongata*; (**F**) An eradicate *I. elongata* colony (white arrow) by a rope; (**G**) *Farrea* sp. growing on a lost rope; (**H**) Polystyrene box; (**I**) A lost fishing pot; (**J**) *Parantipathes larix* and a can lying on the seafloor; (**K**) *Funiculina quadrangularis* and *P. larix* near a lost trammel net in the working position; (**L**) A lost trammel net entangled on rocks; (**M**) A lost trammel net hanging from an outcrop. Scale bar: 15 cm.

Most of the colonies did not host any evident associated fauna. Only one colony was found to be associated with the crinoid *Leptometra phalangium* (Müller, 1841) (Fig. 4G), and some specimens of *Macroramphosus scolopax* (Linnaeus, 1758) were observed hiding and swimming near the coral branches of two colonies (Fig. 4H). The presence of epibionts was observed only on the larger and unhealthy colonies (Fig. 8A,B).

Few colonies were damaged, with a low number of broken branches, and 22 dead colonies were eradicated and laid on the seafloor (Fig. 8C–F). Some eradicated colonies were observed within the proximity of the hard substratum and living colonies (Fig. 8D,E). Nevertheless, most of the colonies exhibited healthy development.

The output of the generalised linear mixed-effects model, which was used to explore the correlation between *I. elongata* abundance and seafloor descriptors, showed that slope, depth, and substratum type (sand, mud, boulders on mud, and rocky shoals) were statistically significant variables (*p* < 0.001) (Table S1). The positive sand substratum coefficient (0.909) and the slope coefficient (0.476) in the model suggested a correlation between the increase in slope and the probability of finding *I. elongata* on the soft bottom. This species was indeed frequently recorded and abundant on the muddy bottom of the offshore dives (M3 and M4) in proximity to the sloping rocky escarpment (Fig. 5). The negative coefficients [depth (− 0.99), substratum—boulders on mud (− 1.828), and substratum—rocky shoals (− 1.828)] indicated that an increase in the predictor variable was associated with a decrease in the abundance of *Isidella*, holding all other variables constant (Table S1).

### Environmental drives

**Figure 9:**
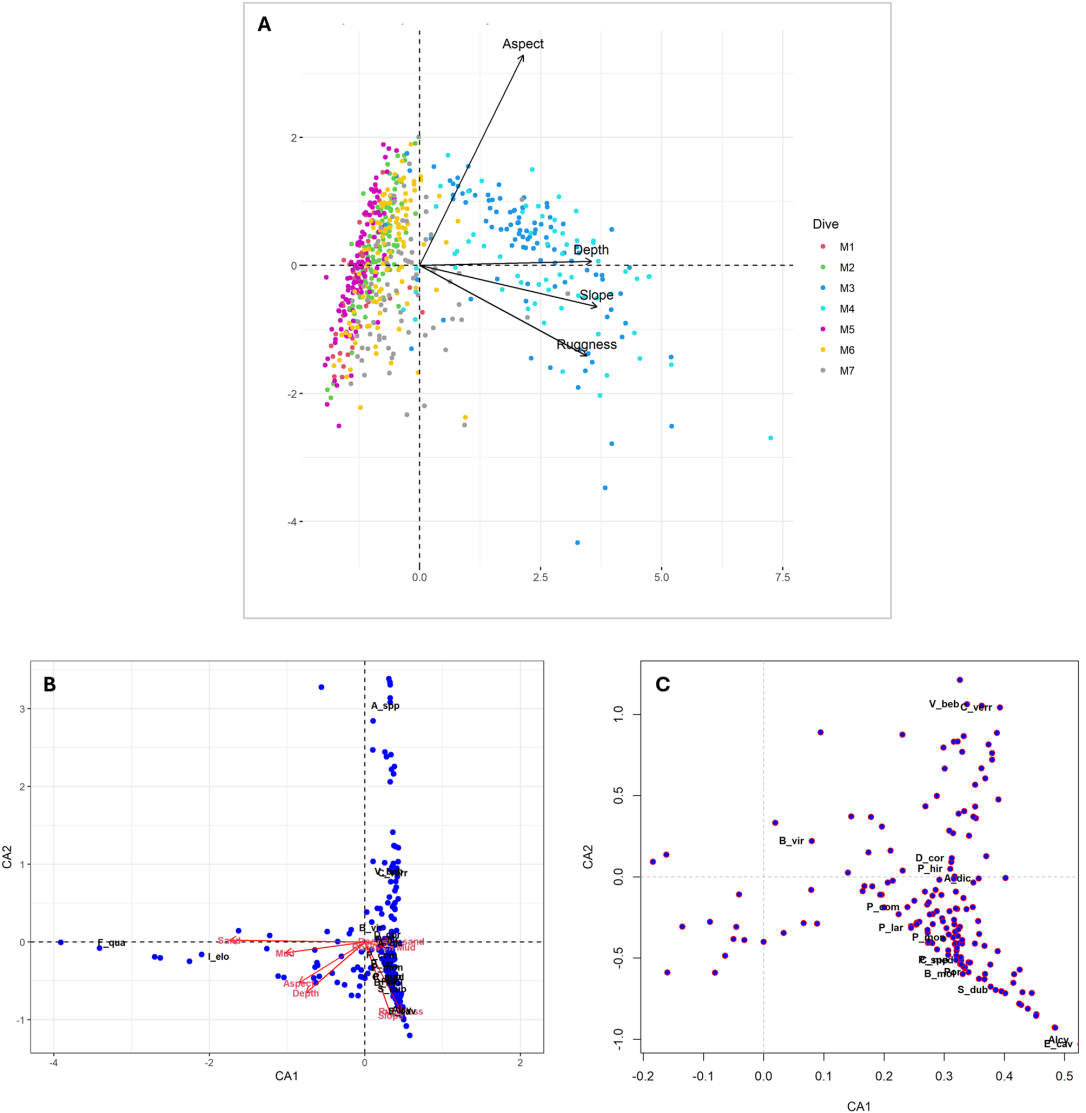
Plots of the multivariate analysis. (**A**) Principal component analysis (PCA) of seafloor descriptors for each dive; (**B**) Canonical correspondence analysis (CCA). Red arrows indicate environmental variables. Points represent sampling unit ordinations based on species abundance and environmental variable constraints; (**C**) Close-up of the central area of the CCA plot. Por = Porifera; P_spp = *Phakellia* spp.; A_spp = *Axinella* spp.; P_mon = *Pachastrella monilifera*; P_com = *Poecillastra compressa*; Alcy = Alcyonacea; C_med = *Chironephthya mediterranea*; B_mol = *Bebryce mollis*; C_ver = *Callogorgia verticillata*; E_cav = *Eunicella cavolini*; P_hir = *Paramuricea hirsuta*; S_dub = *Swiftia dubia*; D_cor = *Dendrophyllia cornigera*; A_dic = *Antipathes dichotoma*; P_lar = *Parantipathes larix*; F_qua = *Funiculina quadrangularis*; I_elo = *Isidella elongata*; V_beb = *Villogorgia bebrycoides*; B_vir = *Bonellia viridis*.

The first two canonical axes of principal component analysis (PCA) explained 80% of the total variance (Fig. 9A). The PCA ordination showed that roughness, depth, and slope were highly correlated, while they were independent of aspect. Additionally, in the PCA plot, the M3 and M4 dives were characterised by depth, slope, and roughness (Fig. 9A; S2), unlike the other dives.

Canonical correspondence analysis (CCA), used to assess how environmental variables may influence the distribution and composition of megabenthic communities, highlighted how *Axinella* spp. were found mainly in southern and shallower dives (i.e., M1 and M2) and that *F. quadrangularis* and *I. elongata* were significantly segregated compared with other dives influenced by substratum variables (Fig. 9B,C). Specifically, *F. quadrangularis* was more closely related to sandy substrata, whereas *I. elongata* was more closely related to sandy and muddy substrata.

### Anthropogenic impact

**Figure 10:**
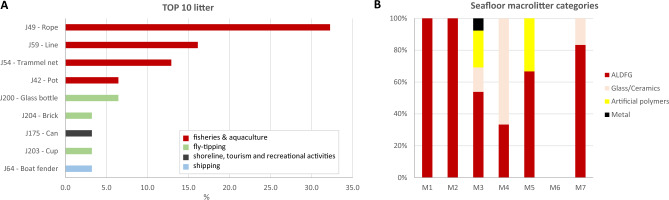
Composition of litter in the study area. (**A**) Top 10 most common litter types according to the Marine Strategy Framework Directive Joint List; (**B**) Percentage frequency of litter macro categories in each site.

A total of 31 litter items were recorded. The dominant litter (80.6%) was made of artificial polymers; however, 67.8% of the items were related to fishing activities (Figs. 8, 10). Ropes (33%), lost lines and bottom longlines (17%), trammel nets (12%), and pots (6.5%) made up the most significant portion of the litter, followed by glass bottles (6.5%). Many long trammel nets were lying on the bottom for several metres. No trawl marks were observed.

The average litter density ranged between 0.0 and 2.0 items 100 m^−2^. Only 13% of the total litter items were associated with sessile invertebrates. Entanglement affected only 2 colonies of *I. elongata* and 5 of the largest colonies of *A. subpinnata*. Unidentified bryozoans, sponges, and bivalves were settled on dead parts of these colonies. No litter used as a refuge by vagile fauna was observed. Fishing-related litter was almost completely covered by epibionts: hydroids were always recorded, followed by high occurrences of sponges (Fig. 8G) and bryozoans.

## Discussion

This study focused on the rich mesophotic circalittoral and upper bathyal coral assemblages observed on the western side of the Asinara Island MPA, increasing knowledge on the distribution of VMEs in the Mediterranean Sea.

This exploration has allowed for the first time reporting on a large *facies* of *Isidella elongata* in the study area, where until recently, only shallower coralligenous reefs were recorded^40,41^.

*Isidella elongata* dwelled on the narrow muddy seabed between shallow coralligenous outcrops on the continental shelf and the rocky slope. The population of this species in the study area was characterised by a high density of colonies (up to 8 col. m^−2^), representing one of the richest *facies* recorded in the Mediterranean Sea within relatively shallow depths (110–298 m). Bo et al.^13^ reported the presence of *I. elongata* with healthy, tall (up to 36 cm), highly branched colonies, and an average density of 0.5 ± 0.04 col. m^−2^ and maximum values of 2.7 col. m^−2^. These density values are comparable to those found in this study and with those reported by Grinyó et al.^42^ for bathyal soft bottoms in the Alboran Sea, where *I. elongata* reached a maximum density of 7 col. m^−2^. Other data from the Mediterranean Sea acquired by ROV-imaging showed that in the Balearic Sea, different values of density were reported when comparing untrawled and trawled bottom areas: 0.23–0.27 col. m^−2^ and 0.0053–0.0062 col. m^−2^, respectively^21^. Similar differences were observed by Pierdomenico et al.^7^ in the Gioia Tauro canyon in the southeastern Tyrrhenian Sea. These authors found a mean density of 0.05–0.4 col. m^−2^, and a maximum density of 1.6 col. m^−2^, with a greater percentage of dead and small colonies occurring in trawled areas. In the central Tyrrhenian Sea, density values of 0.13 ± 0.03 col. m^−2^ were reported by Ingrassia et al.^43^ at Ventotene Island. Along the Catalan coast trawling bycatch data showed density values of ~ 1 col. 50 m^−2^^3^.

The bathymetric distribution of the observed species in the present study is in line with previously published data from other Mediterranean areas poorly impacted by trawling (i.e.,^12^), such as southwestern Sardinia^13,44^. Depending on the specific characteristics of each area, *I. elongata* has been observed at depths ranging from 115 to 1656 m^3,4,10,23^ in the central and western Mediterranean Sea and at depths between 126 and 1125 in the Aegean Sea^11^, with large populations of this bamboo coral predominantly found below 500 m depth^4,11,43,45^. In the past, *Isidella elongata* was known to be present at shallower depths^28,46^, but increased fishing pressure may have induced loss of habitat for this bamboo coral species at that depths^14^. The fishing activity of the trawler fleets around the study area is more concentrated at bathyal depths along the southern western coast of Asinara Island and on the continental shelf in the Gulf of Asinara (i.e.,^39^; Fig. S1). Consequently, the explored area is likely protected from trawling activity because of weather conditions, characterised by the predominance of a strong north westerly wind, the Mistral, which allows fishing activities for only a few days a year and, mainly, the rugged morphology of the seafloor. Indeed, it is surrounded by rocky outcrops and boulders, naturally preserving the bamboo coral *facies*^5,7,13,47^. Therefore, *I. elongata* may be occur on the western seafloor of the Asinara Island MPA even over a wider bathymetric range than that covered by this study, considering the low impact of trawling in this study area. This observation confirms the provisional distribution model proposed by Carbonara et al.^14^. Overall, the populations appeared to be in a good conservation status, and partial necrosis was present in less than 0.7% of the observed colonies. Several colonies are also observed in proximity to hard substrata covered by abundant sediment. According to Pérès and Picard^15^, *I. elongata* is associated with compact mud. Further studies are needed to clarify whether potential differences in the physical, geological, and geochemical characteristics of sediments (e.g., grain size, porosity, water content, composition, and organic content) can contribute to driving the distribution of *Isidella facies*^42^.

Most of the observed colonies were small and likely affected by fishing disturbance. The presence of fishing litter was indeed observed in the investigated area. Most of the observed litter came from artisanal fishing activities and fly-tipping. Even if the presence and abundance of litter and abandoned, lost, or otherwise discarded fishing gear (ALDFG) are lower than those in other Italian and Mediterranean areas^48–50^, some entanglement phenomena, causing physical damage to arborescent benthic organisms have been recorded. However, the number of entanglement events was overall very low, and a few colonies were involved, although the affected species are of considerable conservation interest (i.e., *A. subpinnata*; *I. elongata*). Moreover, a few colonies of *I. elongata* (~ 1%) were completely eradicated, with the rooted holdfast extracted from the surface sediment (Fig. 8C–F). Due to its branched fan shape and lower flexibility, this species can be easily snagged by fishing gear (both fishing lines, trammel and trawling nets)^3,10^. The vulnerability of this bamboo coral as well as other arborescent habitat-forming species to artisanal fishing gear is well documented^10,11,50^. Conversely, sea pens, such as the monopodial *F. quadrangularis*, have greater flexibility and consequently a lower vulnerability to artisanal fishing stress.

Nevertheless, the near absence of trawling in the area has allowed indirect preservation of some bottom enclaves where coral gardens occur, as reported for other Mediterranean sites^10,13,51,52^. These enclaves also encompassed sea pen (e.g., *F. quadrangularis*), black coral (e.g., *P. larix* and *A. dichotoma*), and gorgonian (e.g., *C. verticillata* and *P. hirsuta*) aggregations. The tall sea pen is also primarily threatened by the direct and indirect effects of trawling^7,53,54^. The density values of *F. quadrangularis* found in this study are comparable to or even greater than those reported in the literature; for example, 0.83 col. m^−2^ in the Adriatic Sea at a depth of 162 m^53^ and from 54.7 to 7771.6 ind. km^−2^ in the northern and central Adriatic Sea^54^; 0.05–0.35 col. m^−2^ mean density along the Gioia Canyon in the southern Tyrrhenian Sea^7^; 0.00–0.08 col. m^−2^ in the Ionian Sea^55^; and 0.2 col. m^−2^ in the Ligurian Sea (Portofino)^56^. Aggregations of this sea pen in the Mediterranean Sea are typical of hydrodynamically active areas such as the bases of ridges, slightly sloping escarpments, and continental slopes^57^. *Funiculina quadrangularis* is generally found on viscous mud with a very fluid superficial layer (~ 250–500 m depth, sensu Pérès and Picard^15^), and has slightly different distribution patterns than *I. elongata*, likely due to the differences in edaphic feature preference and sensitivity to the impact of fishing activities. However, in the study area, the two species exhibited almost the same distribution. The presence of *P. larix* on the boulders is also relevant. This species is one of the least well-known black corals from an ecological point of view^58^. It may reach impressive abundances and sizes, forming important *facies* in the deep-sea realm in high topographic structures (i.e., seamounts, rocky ridges, and rocky shoals) subjected to complex hydrographic regimes, enhancing the resuspension of nutrients and the settling of large filter-feeders^59,60^. In the last decade, ROV surveys have increasingly shown scattered colonies of this monopodial or sparsely branched species in the Mediterranean Sea, both along the continental shelf and in deep waters (up to 2300 m)^58,61^. However, dense populations are rarely reported in the literature for the Mediterranean basin^58^. In this study, this black coral was abundant and healthy, but its density was lower than that recorded by Bo et al.^58^ on Montecristo Island, where *P. larix* reached a maximum density of 2.3 col. m^−2^.

Different geomorphological and environmental factors and trophic characteristics of the habitat are known to influence the distribution of coral species^3,11,14,43^. The main drivers of *I. elongata* distribution in this study were depth, slope, and type of substratum. It was very abundant in the dives characterised by consolidated/semiconsolidated mud at greater depths, which are conditions typical of its habitat, and with increasing slopes. Some authors^3,28,46^ have also observed a preference of these populations for deeper seabeds and steeper slopes, as a consequence of intense removal of *I. elongata* colonies by trawling on shallower and flat ground. Conversely, black coral species were typically found in areas characterised by steeper slopes and rocky substrata.

Although depth is considered the main factor influencing the distribution and density of marine species^45,62^, in this case, the distribution of *I. elongata* seems more strongly linked to other factors, such as natural protection from trawling.

*Isidella elongata* plays an important ecological role in the bathyal environment of the Mediterranean Sea because its three-dimensional habitat creates biodiversity hotspots, providing refugia and secondary biological hard substrata for several fish and invertebrate species^3,21^. In this regard, only one colony of *I. elongata* was associated with the crinoid *Leptometra phalangium*, and *Macroramphosus scolopax* specimens hiding between its branches were observed for only two colonies. None of the other commonly associated species (e.g., *Amphianthus dohrnii*, and *Anamathia rissoana*) were observed, probably due to the shallow depth range where these colonies were found. Moreover, the high abundance of smaller colonies, which may not be large enough to sufficiently attract spawners or juveniles of several species, should also be considered.

Several species were recorded in this study, and most of the anthozoan species are of significant ecological and conservation importance. These are listed in the IUCN Red list as ranging from vulnerable to critically endangered (Table 2), which is the maximum risk category before extinction. The purpose of this list is to provide decision makers with a legal instrument for species conservation and ongoing management of the Mediterranean Sea^63^, although it does not represent a protection legal mandate^64^.

The finding of bamboo coral *facies* associated with precious black coral forests, along with other recent discoveries of rhodolite beds and coralligenous assemblages^41^ off the western Asinara Island MPA, highlights the important need for further protection measures in the area.

Bamboo coral and black coral gardens serve as valuable long-term biological archives^13,65^, but they have very slow recovery abilities. The impact of fishing activities can indeed strongly threaten their preservation. As has already been stated for precious red coral populations, pristine coral ecosystems can currently be considered rare in the Mediterranean basin^58,66^.

The natural fragmentation of the populations, slow growth rates, limited larval dispersion and population connectivity, late maturity age, very long impact recovery period, and susceptibility of the colonies to destruction are all factors that can limit the recovery of colonies if subjected to strong and/or continuous stresses^13,67^.

Although recent investigations have more frequently revealed the presence of these assemblages than in the past, they have also shown the fragility of these ecosystems and the detrimental effects of human-related activities on habitat complexity and biodiversity^50^.

The impacts of fishing activities and marine litter on these ecosystems, for example, represent an acknowledged environmental issue, particularly considering the increasing demands for the conservation of biodiversity. Recently, the Food and Agriculture Organisation^36^ recommended the establishment of protected areas where VMEs occur, introducing regulations and adopting actions to manage fisheries in deep-sea ecosystems. Moreover, the European Biodiversity Strategy for 2030 mandates the establishment of new protected areas to expand the European protected marine network (Natura 2000).

These VMEs, which play a key role in both homogenous muddy and rocky environments, supporting biodiversity, and providing ecosystem services have experienced a decline (low densities of habitat-forming species and impoverishment of habitat complexity and diversity) in the Mediterranean Sea since the 1970s due to intensive fishing activities and other human pressures. Considering all these aspects, in addition to the low recovery rates of most species characterising the observed assemblages, it is suggested to enlarge the perimeter of the Asinara Island MPA to include these vulnerable benthic assemblages. The protection of these coral gardens in the study area could facilitate the effective preservation of the ecological function of these key coral species, which are relatively unimpacted by anthropogenic stressors. However, transdisciplinary studies, participatory processes addressing both protection and social goals, and stakeholder support are required to achieve an effective MPA and marine biodiversity conservation^68,69^.

## Materials and methods

### Data acquisition

The study area is located approximately 3–6 km from the western sector of the Asinara Island MPA (northwestern Sardinia, NW Mediterranean Sea), in water depths ranging between 66 and 307 m (Fig. 1). The continental shelf has an average length of 6.5 km and a slope of 1°–2°.

In the summer of 2022, a multibeam and ROV survey was carried out onboard the R/V VEGA 1 during the research cruise ‘‘MoRinet”. Geographic and bathymetric data from the study area (20 m horizontal resolution), which are available in the literature (MArineGeohazard Along the Italian Coasts (Magic) project^70^), were used to support the investigations. High-resolution bathymetric data were collected at seven sites (M1–M7, Fig. 1), using an EM 2040C multibeam (Kongsberg) operating at frequencies of 200–400 kHz and controlled by the seafloor information system (SIS) acquisition software. The multibeam survey was performed at a frequency of 300 kHz with a beam angle of 55° and an average sailing speed of approximately 4.5 knots. Analysis of bathymetric and backscatter data was carried out using CARIS HIPS and SIPS software, while morphological analysis data were processed using a geographic information system (GIS, ArcGIS 10.1).

Video observations were collected using a Pollux III (GEI) ROV system equipped with a digital camera (Sony CCD 1/3″), a high-definition camera (SonyHDR-HC7), three 15 cm scale laser beams, and an underwater acoustic tracking positioning system, Ultra-Short Baseline (USBL) (Linquest Tracklinq 1500 MA), which acquired position signals every 2 s, with up to 0.25° accuracy. Specific attention was given to maintaining a constant ROV cruising speed of approximately 0.5 knots and an altitude of approximately 1.0 m from the bottom. The ROV performed eight transects from 400 to 900 m in length, recorded in both high-resolution (HD) and low-resolution modes. Except for the Fp dive, all other dives were performed in proximity to the sites explored with multibeam (M1–M7, Fig. 1).

### Data processing

A total of 6 h and 53 min of ROV footage were recorded, covering a linear distance of ~ 5 km. The videos were processed using the freely available internet software VLC.

The substratum types along the transects were visually classified into four categories: detritic sand, mud, boulders on mud, and rocky shoals.

To characterise the megafaunal diversity of the sites, all megafaunal taxa visible along the video transects were classified to the lowest possible taxonomic level, considering the morphological diagnostic characters reported in the available literature and comparing the images to photo guides (i.e.,^8,71^; and references therein).

The smooth plot of the georeferenced ROV transects was imported into the GIS software ArcGIS v10.3.1. The portions of the video not relevant (i.e., ascent and descent ROV phases, ROV far from the seafloor, recording close-up images, and frames with poor visibility or out of focus) were not considered in the analyses. Each cleaned ROV video transect was divided into 5 m long sampling units (SUs; 5 m long and 1 m wide; estimated from the laser beam distance), according to previous studies of Pierdomenico et al.^7^, Enrichetti et al.^72^, and Grinyó et al.^42^. All the observed colonies of *I. elongata* were counted in each SU and divided into two size classes, using laser beams as a metric scale, following Pierdomenico et al.^7^ and Ingrassia et al.^43^: small colonies (< 20 cm height) and large colonies (> 20 cm height). To detect the population structure of *I. elongata*, the height of colonies was measured by Image J software on a subsample of colonies (~ 25% of total colonies) randomly selected from colonies perpendicular to the camera and coplanar with laser beams at all sites. The presence of epibionts or necrosis was reported, as was the presence of dead colonies.

In addition, target charismatic structuring species, namely, the scleractinian *Dendrophyllia cornigera*; the gorgonians *P. aramuricea hirsuta*, *Callogorgia verticillata*, and *Viminella flagellum*; the sea pen *Funiculina quadrangularis*; the antipatharian *Antipathes dichotoma*, and *Parantipathes larix*; were also counted. For each target species, the occupancy in the SUs (%) was estimated, and the average density of coral colonies (no. of colonies m^−2^ ± standard error) was calculated by dividing the total number of coral colonies counted for the estimated video coverage area of each SU.

The presence of benthic litter and abandoned, lost, or otherwise discarded fishing gear (ALDFG), along with their interaction with species was recorded, following the methods described by Angiolillo et al.^73^.

### Statistical analysis

The environmental parameters and species distributions were statistically analysed via multivariate analysis using R software^74^. To avoid noise in the final outputs, the dataset excludes the fish fauna and all taxa for which less than 1% of colonies or individuals were found. Moreover, the dive Fp was excluded from multivariate analysis because multibeam data were not available for the Fp site.

The environmental variables used included depth, substratum type, and seafloor descriptors [i.e., slope, terrain roughness (referred to as Ruggness in the analysis), and aspect (referred to as Aspect in the analysis)] derived from high-resolution bathymetry.

A generalised linear mixed-effects model with a Poisson distribution was applied to the *I. elongata* data using the lme4 package in R. This model allows us to analyse species abundance in relation to environmental variables and to assess the random effects of dives. This modelling approach accounts for both fixed effects (environmental predictors) and random effects, which capture the variability due to unmeasured factors such as differences between dives. A comprehensive preliminary analysis, including residual analysis and QQ plots, was conducted to ensure data suitability and model assumptions.

PCA was performed using the ade4 package of R software. This analysis, based on dimensionality reduction, was used to explore and identify patterns and correlations among the variables. To represent the distribution of seafloor descriptors for each dive, boxplots were generated to provide a visual summary of the interquartile range (IQR), median and outliers, allowing for a clear description of the variation in environmental conditions across the sampled locations.

Finally, a CCA was used to analyse how variations in species composition were associated with environmental gradients^75^. The analysis was conducted using the vegan package of R software.

### Supplementary Information

Supplementary Information.

## Data Availability

All the data generated or analysed during this study are included in this published article (and its Supplementary Information files). The datasets generated are available from the corresponding author upon reasonable request.
