# Supplementary material for: Healthy assemblages of Isidella elongata unintentionally protected from trawling offshore of Asinara Island (northwestern Sardinia, NW Mediterranean Sea)
Source: Sci Rep. 2024 Jun 4;14:12813. doi: 10.1038/s41598-024-63652-1 (PMC11150251; doi:10.1038/s41598-024-63652-1)
Supplement: Supplementary file 1 — Supplementary Information. [file 41598_2024_63652_MOESM1_ESM.docx]

**Supplementary materials**

**Healthy assemblages of *Isidella elongata* unintentionally protect from trawling offshore the Asinara Island (northwestern Sardinia, NW Mediterranean Sea)**

Angiolillo M^1*^, Di Lorenzo B^1^, Izzi A^1^, Giusti M^1^, Nonnis O^1^, Pazzini A^1^, Trabucco B^1^, Tunesi L^1^

^1^ Istituto Superiore per la Protezione e Ricerca Ambientale (ISPRA), Via Vitaliano Brancati, 60, 00144, Rome, Italy

^*^ michela.angiolillo@isprambiente.it


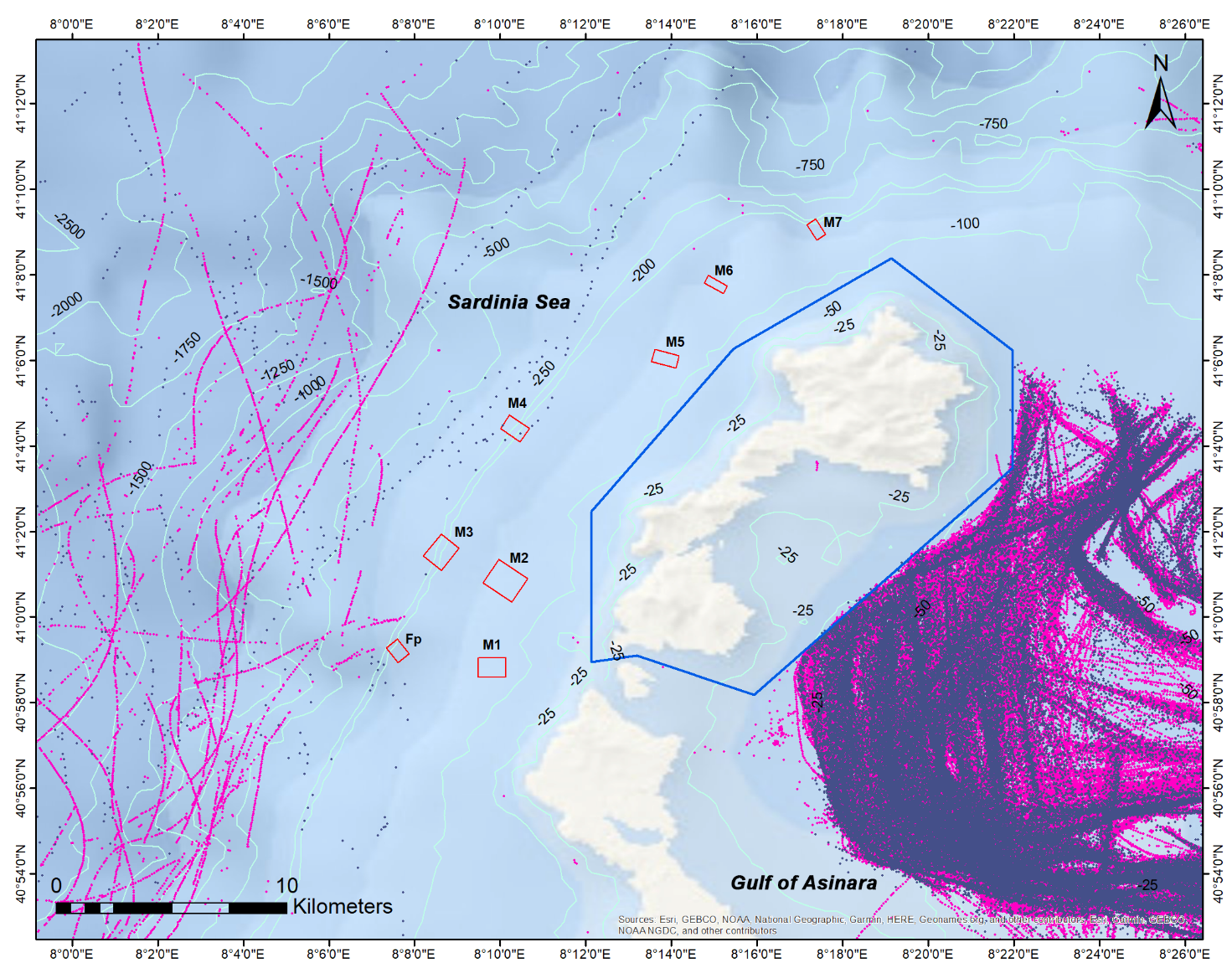


**Figure S1.** Trawling activity around Asinara Island marine protected area (MPA), based on Automatic Identification System (AIS) data classified as "fishing" through speed analysis (G. Franceschini). It shows the absence of trawling in the study area where *Isidella elongata* facies were observed. The grey and magenta dots refer to 2019 and 2020 fishing data, respectively. The red rectangles indicate the sites where multibeam and ROV surveys were carried out. The blue polygon represents the perimeter of Asinara Island MPA. The Italian Ministry of infrastructure and transport kindly provided AIS data as part of a collaboration within the Marine Strategy Framework Directive. Background bathymetry data were obtained from EMODnet (<https://emodnet.ec.europa.eu/en/bathymetry>). The map was generated using ESRI's ArcGIS 10.3 for Desktop software ([http://www.esri.com](http://www.esri.com/)), version 10.3.0.4322.

**Table S1**

Output summary of generalized linear mixed-effects (GLMM) model.

| **Fixed effect** | **β** | **SE** | **z value** | **ρ** |
| --- | --- | --- | --- | --- |
| (Intercept) | -0,697 | 0,546 | -1,277 | 0,202 |
| Ruggedness | 0,007 | 0,067 | 0,101 | 0,920 |
| Slope | 0,476 | 0,071 | 6,672 | **<0.001** |
| Aspect | -0,022 | 0,048 | -0,451 | 0,652 |
| Depth | -0,990 | 0,104 | -9,507 | **<0.001** |
| Substratum - boulders on mud | -3,253 | 0,425 | -7,646 | **<0.001** |
| Substratum - rocky shoals | -1,828 | 0,244 | -7,503 | **<0.001** |
| Substratum - sand / mud | 0,909 | 0,149 | 6,099 | **<0.001** |
| **Random effects:** | **Variance** | **Std.Dev.** |  |  |
| DIVE | 1.945 | 1.395 |  |  |


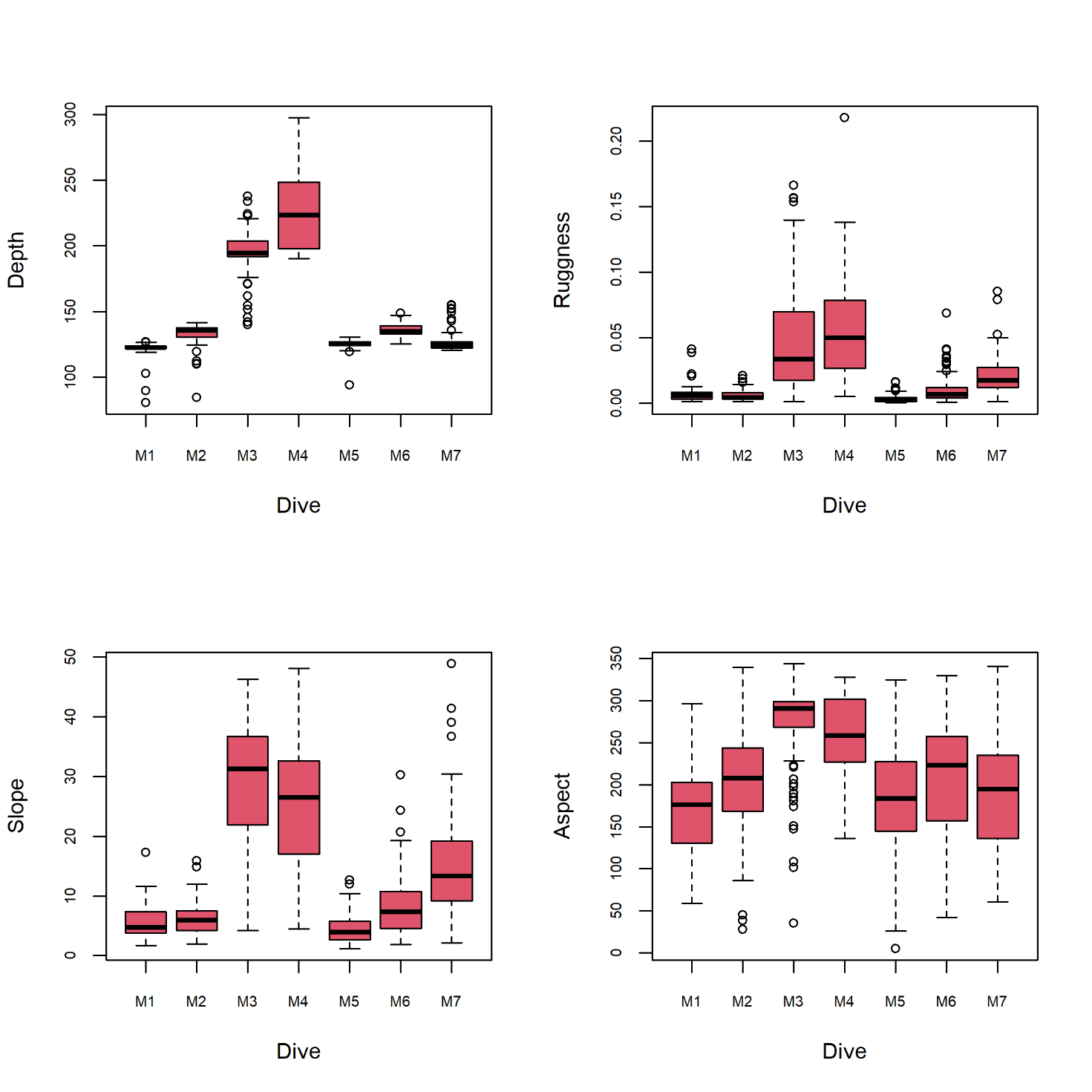


**Figure S2.** Boxplot representation of seafloor descriptors for each dive.
